# Micro/Nanopatterned Superhydrophobic Surfaces Fabrication for Biomolecules and Biomaterials Manipulation and Analysis

**DOI:** 10.3390/mi12121501

**Published:** 2021-11-30

**Authors:** Marco Allione, Tania Limongi, Monica Marini, Bruno Torre, Peng Zhang, Manola Moretti, Gerardo Perozziello, Patrizio Candeloro, Lucia Napione, Candido Fabrizio Pirri, Enzo Di Fabrizio

**Affiliations:** 1 Center for Sustainable Future Technologies @POLITO, Istituto Italiano di Tecnologia, Via Livorno 60, 10144 Turin, Italy; fabrizio.pirri@iit.it; 2 Dipartimento di Scienza Applicata e Tecnologia (DISAT), Politecnico di Torino, Corso Duca degli Abruzzi 24, 10129 Turin, Italy; monica.marini@polito.it (M.M.); bruno.torre@polito.it (B.T.); lucia.napione@polito.it (L.N.); enzo.difabrizio@polito.it (E.D.F.); 3 Biological and Environmental Science and Engineering (BESE) Division, King Abdullah University of Science and Technology (KAUST), Thuwal 23955-6900, Saudi Arabia; peng.zhang@kaust.edu.sa (P.Z.); manola.moretti@kaust.edu.sa (M.M.); 4 BioNEM Laboratory, Department of Experimental and Clinical Medicine, Campus S. Venuta, Magna Graecia University, Germaneto, Viale Europa, 88100 Catanzaro, Italy; gerardo.perozziello@unicz.it (G.P.); patrizio.candeloro@unicz.it (P.C.)

**Keywords:** superhydrophobic surfaces, micro/nanofabrication, biomolecules

## Abstract

Superhydrophobic surfaces display an extraordinary repulsion to water and water-based solutions. This effect emerges from the interplay of intrinsic hydrophobicity of the surface and its morphology. These surfaces have been established for a long time and have been studied for decades. The increasing interest in recent years has been focused towards applications in many different fields and, in particular, biomedical applications. In this paper, we review the progress achieved in the last years in the fabrication of regularly patterned superhydrophobic surfaces in many different materials and their exploitation for the manipulation and characterization of biomaterial, with particular emphasis on the issues affecting the yields of the fabrication processes and the quality of the manufactured devices.

## 1. Introduction

Superhydrophobicity is the term used to indicate the peculiar property of some surfaces to repel water with extreme effectiveness and it has been understood for decades that the phenomenon appears when the intrinsic repellent properties of a material are combined with the deviation of its morphology from the bare flat profile. The effect has been known for a long time, as many examples can be found in nature [1]. The leaves of Lotus flower are the most famous of them, such that the phenomenon is frequently named the “Lotus effect” [2]. The reason nature developed such surfaces is for the need of some biological systems to keep dry under the rain and, in many demanding environments, to exploit self-cleaning. It has been demonstrated that the combination adhesion of water at the surface and capillary forces on the dust results in an effective and fast cleaning of the surface, with no need of any additive or detergent. This property has been largely studied from the perspective of its possible application on biomimicking surfaces with self-cleaning properties, with particular interest for applications in the field of photovoltaics, where efficiency is strongly affected by surface cleanness and transparency, with the obvious advantages in this type of tools [3,4,5,6]. A great deal of effort has further been devoted to the development of superhydrophobic surfaces (SHSs) for low-friction applications in mechanical and fluidodynamical systems [7,8,9,10]. Similarly, due to their peculiar properties, SHSs have been extensively studied for a wide range of applications [11,12,13,14,15,16,17].

From the very early stage of the research on superhydrophobicity, one of the most promising fields of application has been foreseen in biology and medicine [14,18,19,20,21,22,23,24,25,26,27,28,29]. A large amount of literature has been produced to study the development of SHSs for applications devoted to patterning with superhydrophobic coatings several biomedical instruments such as catheters or endotracheal tubes. These have the final purpose of both decreasing the fluid friction in the device to avoid clogging of tubes and apertures, and, at the same time, decreasing bacterial adhesion to limit biofouling when in contact with blood or bodily fluids [30,31]. These coatings are frequently based on disordered self-assembled micro/nanostructures that contemporarily diminish fluid friction and provide antiseptic treatment with improved liquid transport properties [32,33,34,35]. Among these activities, a special mention is deserved for the studies on the specific case of surfaces aimed at improving interaction with blood, to avoid blood cell disruption for devices to handle blood in a liquid state. This subfield of research has received a lot of attention and is already the object of specifically focused reviews [22,36,37,38]. A similar application is the one intended to develop superhydrophobically coated medical devices for drug delivery [39,40,41,42,43,44,45].

Different types of applications are represented by those including lab-on-chip devices. Among them, we can mention the microfluidics-related applications, where the interest in SHSs is in the capability to implement superhydrophobic coating in microfluidics devices to control liquid flow and mixtures. These SHSs are typically based on disordered and, frequently, self-assembled coatings, even if examples of coated microfluidics devices based on ordered pillars or microstructures do exist.

The main group of these lab-on-chip applications is then represented by those devices exploiting the capability to create or control drops of liquid by reducing the interaction with the underlying substrate. This leads to the creation of disposable microfluidic diagnostic devices, where the SHS supports drops of analytes and facilitates their flow towards active areas, controlling biomolecules/cellular deposition to realize microanalytical/cellular microarrays or engineered tissues.

This review will particularly focus on SHSs applications, which allow controlling the deposition of biological material for analytical applications, also making use of the dynamics of liquid movement inside the drop or of the drop with respect to the substrate, relying on regularly patterned structures. In particular, we will mainly focus on the fabrication issues raised by this type of devices, on the different ways they have been, or could be fabricated and how the different options are more favorable for some kinds of applications rather than others. In the following of this paper, after a short introduction to the phenomenon of superhydrophobicity (in Section 2), we will move to a general introduction to the most used fabrication techniques for realizing these devices (Section 3), with a specialized section to deal with the problem of the surface coating of superhydrophobic devices (Section 4). We will then detail the main examples reported in literature either for the manipulation of deoxyribonucleic acid (DNA), proteins, and peptides (Section 5), or the manipulation of cells and cellular materials, including the organized cells growth (Section 6), before moving to few concluding remarks.

## 2. Phenomenology of Superhydrophobicity

Since we are going to use the phenomenology related to superhydrophobicity to describe the applications, we are going to provide a short introduction on the physical origin of the phenomenon and its most important peculiar aspects.

It is an everyday experience that when water wets a solid surface, the liquid residuals tend to split into drops. If small enough, i.e., when gravity plays a negligible role on their profile, these parcels of water tend to assume the shape of a truncated sphere, which is due to the interplay of surface tension at the interfaces and the capillary forces in the liquid. Its contact angle on the underlying surface, i.e., the angle of the tangent to the sphere at the contact line with respect to the plane, is determined by the surface energy balance between the liquid–surface, the surface–air, and the liquid–air interfaces [46].

Such contact angle can vary a lot depending on the substrate and on the different types of liquid. Importantly, it has been recognized that it is solely a surface property and does not depend on the bulk properties of the underlying material. A particularly remarkable example is the case of graphene: it has been demonstrated that it can be “transparent” to wetting when its thickness is decreased down to a single monolayer, i.e., a water drop on a graphene-monolayer-covered surface shows about the same contact angle as the pristine material [47]. Nevertheless, it turns out that this angle changes as far as the number of graphene layers increases and it converges rapidly to the contact angle value of bulk graphite as soon as the graphene layers reaches few units. Basically, the “memory” of the presence of the underlying bulk material is lost after around one nanometer or less of distance.

By definition, a surface whose contact angle with water is larger than 90° is usually qualified as hydrophobic, while it is hydrophilic in the opposite case. Analogous definitions can be formulated for other types of liquid. For the case of nonpolar ones, we can generically talk about oleophobicity, even if some authors make some more distinction between them, based mainly on the type of oil and its physical properties [16]. Moreover, a surface can be selectively hydrophobic or oleophobic, or it can be both. In this case, authors speak about amphiphobicity or omniphobicity, but we will not argument about the distinction between these two definitions. In fact, despite the large interest raised by oleophobicity and its combination with hydrophobicity for its fundamental distinctive properties and for potential possible applications, we are focusing on the systems that find applications in the manipulation of biomaterials, which are typically prepared in water or water-based physiological solutions. For this reason, in the following of this review, we will completely focus on the water case, keeping in mind that similar conclusions are applicable to different types of liquids and oils, while more general discussions on possible applications can be found in other specific reviews [16].

**Figure 1:**
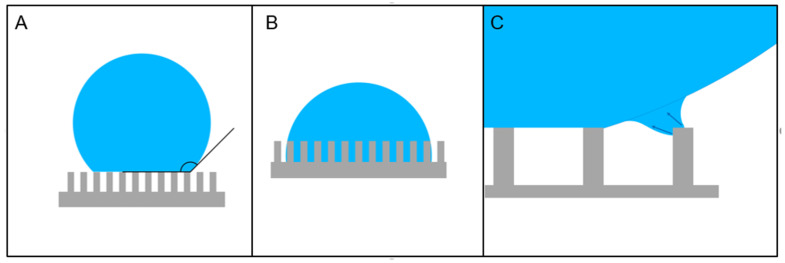
Schematic representation of the Cassie–Baxter (**A**) and Wenzel (**B**) states of a drop of liquid on a patterned surface. Panel (**C**) depicts a schematic representation of the process or regression of the liquid in the Cassie–Baxter state upon water evaporation. The volume reduction pulls away the drop from the outmost pillars in the structure creating a deformation of the water surface from the perfect spherical shape. Such deformation becomes energetically unfavorable once the water protrusion becomes too long, the liquid then flows away from the pillar completing the drop jump to the next inner stable state. This process creates a liquid shear flow from the abandoned pillar to the neighbor one. This process is at the basis of some of the applications used by several authors described in this review to stretch different materials across gaps in superhydrophobic surfaces (SHSs).

The superhydrophobic behavior results from the combined interplay of two factors: (1) the intrinsic hydrophobicity of the surface and (2) its structure. While on a flat surface, water contact angle usually does not exceed 120°, even for the most hydrophobic materials [48]; the introduction of surface roughness can further increase this value. The mechanism behind the evolution toward superhydrophobicity was initially studied many decades ago, leading to the classical theory where two different regimes were identified, named after the authors who first made these studies (Figure 1). The Wenzel model describes the case in which the droplet completely wets the rough surface from the tip to the hollows. The increase in the contact angle due to roughness is explained using a simple geometrical approach, by correcting the balance in the surface energies equation with a dimensionless parameter, given by the ratio of the actual surface area and its projection on the plane over which the drop lies [49]. In this model, the drop still sits on the whole surface with just a modification of its contact angle due to a geometric rebalancing of the surface energies due to the roughness. A different situation is the one described by the Cassie–Baxter model [50]. This second scenario describes a situation in which the drop no longer sits on the bottom of the surface but is suspended over the tips and the most prominent parts of the solid surface, while it does not touch the bottom of the deepest point and valleys. In this second case, the air trapped between the drop and the surface increases the contact angle. To see it in another way, the roughness of the surface is so pronounced that, combined with surface hydrophobicity, it repels water from the deepest wells and valleys of the substrate. This happens because in these areas, the contact surface between the liquid and the surface results so large to make more energetically favorable the configuration in which the drop “floats” over spaces filled by air.

Even if the phenomenon can be found in nature and in principle can be obtained on simply roughened surfaces, in view of application, it can be convenient to use manufactured surfaces that couples increased surface area with oriented patterns to align and orient the droplet deposition. This has the obvious advantage of getting, in such a way, a surface with tunable roughness, which can be easily designed and manipulated to obtain the required contact angle values, as well as specific structures to concentrate or to pin the drop at specific positions. This allows the control and fine manipulation of the drop for specific applications, since positioning a drop over a completely and uniformly superhydrophobic surface can prove difficult. It is well-known that any massive body on a surface usually experiences a resistance to lateral movement due to adhesion. From this consideration, we can derive the usual definition of the adhesion coefficient ν, which is given by the ratio of the lateral force needed to move the body and the normal force to the interface. If the surface is slowly tilted, there will be a particular angle at which the body starts moving away, sliding on the flat surface. It is obvious to see that this critical angle α is related to the friction parameter by the relation ν = tan(α). SHSs in the Cassie–Baxter state are characterized by a very small adhesion constant, due to the very small fraction of the substrate surface with the liquid. This leads consequently to very small values of α, which is called in this case the roll-off angles: an inclination of the sample above α, or alternatively, a minimal side force, can push the drop to detach and roll away over the surface [51]. While this aspect can be useful for some specific uses such as self-cleaning surfaces, it might be detrimental in applications requiring better control and positioning of the surface. In such cases, the control of surface structure with the introduction of pinning points or roughness gradients to control and limit the drop movement can be extremely useful. In this respect, a controlled surface patterning can give the flexibility in the surface structure required by this kind of application.

A regular array of pillars can assure very high contact angle surfaces when it reaches the Cassie–Baxter state, i.e., when the liquid drop is exclusively in contact with the tops of the pillars and completely suspended from the bottom of the structure. This is achieved for a specific range of the surface coverage *Φ*, which is basically the fraction of the substrate surface that is in contact with the liquid. According to the Cassie–Baxter theory, the effective contact angle *θ*_e_ can be written as

cos*θ*_e_ = *Φ* (cos*θ* + 1) − 1,

where *θ* is the contact angle to the flat surface. Hence, the smaller the *Φ*, the larger the resulting contact angle.

Nevertheless, we cannot decrease *Φ* indefinitely, as this would lead to a collapse of the drop over the pillars in a process frequently called impalement of the drop [52,53]. It is intuitively easy to understand that the condition to maintain the Cassie–Baxter regime and to avoid going to the Wenzel one is strongly dependent on the parameter *Φ*. In the case of the regular pillar array, if the pillars become too small in diameter with respect to their interdistance, each pillar will have to sustain an individual force to retain the liquid at their tips that will be too large for such a small area. The pillars will then penetrate into the liquid and the drop will collapse to the Wenzel regime [54,55].

In general, in the case of a regular array of pillars in the Cassie–Baxter regime, the fraction of surface in contact to the liquid together with the height of the pillars rules the impalement of the drop, the passage to the Wenzel state, the subsequent decrease of the contact angle, and the increased friction of the surface on water. Other parameters such as pillars’ shape and their local arrangement are actually irrelevant. Even if, in general, superhydrophobicity does not require a specific arrangement of the structures on a surface, a regular organization of the pillars is essential when other physical processes are involved (e.g., evaporation), as for the applications we are considering. In the controlled deposition of biomaterial, parameters such as the pillar interdistance and their arrangement become crucial, since it allows either a steady position of the drop during the deposition and a regular movement of the drop borders during the evaporation process. The use of specific patterns, e.g., concentric, can be used to guide the evaporating droplet perimeter along a retraction desired path, while the solution becomes more concentrated. In the same manner, the size of the gap over which the dried material is deposited can be tailored and tuned to obtain free-standing analyte layers between close pillars. The use of specific fabrication processes is able to regulate these parameters [56].

## 3. Fabrication Approaches for the Realization of Ordered SHS

Due to the relatively general character of the phenomenon of superhydrophobicity and the very large tolerance on fabrication geometries and materials with which it can be obtained, there is a relatively large number of techniques that can be used to produce an SHS. Nevertheless, some applications need to restrict to the case of regularly and ordered pattern of structures. This introduces a first general limitation on the possible fabrication strategies.

It is, first of all, necessary to find a method to define the regular pattern on the surface of the material. This can be performed by using the many different technological advances developed in microfabrication during the last decades.

Microfabrication is a well-established technology, currently used worldwide to produce many devices ranging from microelectronic circuits to micro-electromechanical systems (MEMS). The standard processes and techniques of this field of technology can be easily applied to the realization of standardized SHSs, which can benefit from the high throughput and great level of reproducibility that those technologies have achieved. Lithography, in particular, allows the possibility to define patterns tailored for any specific application. Among the types of lithography, the optical one, which makes use of contact or projected masks, is probably the most common and well-established, not only in research but also in industry, being the standard method used in microelectronics and MEMS fabrication. Optical lithography is characterized by the highest throughput thanks to the serial reproduction of a predefined pattern printed on an optical mask, but it lacks versatility, since a different mask is necessary to define every particular pattern.

Differently, maskless-scanning-based methods such as direct laser writing or electron beam lithography can provide more flexibility, even if the latter is usually slower and comparatively much more expensive, leading to lateral resolutions that are in most cases overperforming the needs for the realization of SHS. These last methods are those typically used to prepare the masks for contact and projection optical lithography as well.

Once the pattern on the surface is defined, it is necessary to create the surface profile that leads to superhydrophobicity. Following a very broad and approximated distinction frequently used in the literature of nanofabrication, we can, broadly speaking, divide fabrication techniques into the two so-called bottom-up and top-down groups. The first group indicates the ones relying on the growth or assembly of fundamental constituents from a liquid or gaseous phase on the surface of the sample. The second one indicate the techniques used to dig or engrave a bulk material to realize a pattern of structures by subtraction of the material from the substrate using etching techniques, either based on plasma-assisted etching or chemical erosion forma reagent dispersed in a liquid or gas solution [57].

### 3.1. Top-Down Fabrication Methods

**Figure 2:**
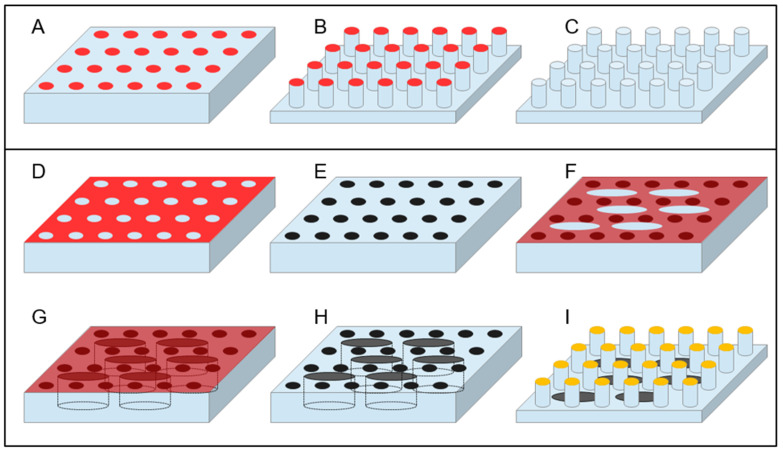
Schematic representation of the most common top-down approaches to realize a patterned device with a three-dimensional (3D) profile. Panels (**A**–**C**) describe the standard etching methods. A substrate (most frequently Silicon but the technique can be used on many different materials) is selectively covered by photoresist or other protective masks to define a regular pattern on the surface (**A**). The substrate is then attacked, usually by plasma etching, to produce the 3D structures (**B**). The top mask is finally stripped to produce a clean patterned surface (**C**). Some authors have proposed the combination of the realization of a 3D structure with the possibility to have holes in the substrate. This process is schematically illustrated in panels (**D**–**I**). The concept is exactly the same, but in this case, two overlapped lithography steps are superposed and aligned. The first one defines a hard mask to create the pillars (**D**–**E**), while the second create a mask to allow the etching of the through-substrate holes (**F**–**G**). After removal of this second protective mask (**H**), the final structure can be realized and, in case a specific surface material is needed selectively on the surface in contact with the liquid, this can be predeposited under the hard masking material placed in step (**E**); after final removal of this mask, the underlying material will appear (**I**).

In this category, all types of etching can be included, both dry—i.e., those making use of a plasma-assisted etching or of an ion milling, and wet—i.e., those using chemical solutions to selectively dissolve a substrate in the regions left uncovered during the lithography process. Among the dry etching processes, Deep Reactive Ion Etching (DRIE) certainly deserves a special mention, due to its capability to drill structures in silicon at basically any desired depth with great control of the etch rate and leaving a controlled slope of the side walls of the realized structures that can reach 90° or more. DRIE is a process that continuously alternates two different steps within an interval of typically 10 to 20 s: A plasma-induced deposition of a perfluorinated organic polymer starting from a gaseous precursor and a fluorine-ion-based reactive ion etching. The key point of the process is that the parameters are tuned in such a way that, while the polymer deposition is a rather isotropic process, the reactive ion etching is more directional, due to the energy impressed to the fluorine ions by the bias induced between the plasma and the sample. Hence, during the etching part of the cycle, while the predeposited polymer layer is strong enough to protect the sidewalls of the structure from ion-induced erosion, the ions bombardment at the bottom surface of the sample removes it efficiently and rapidly. The combined effect of the alternation of the two actions leads to the realization of deep structures [58,59,60,61]. Figure 2A–C illustrates a simple schematics of this process.

The process is so well-established that it has been used already in several implementations of SHS. In the case of silicon, the process is so selective that the etch rate is very fast and the rate ratio with respect to any masking material (i.e., the ratio of the etch rate on silicon with the corresponding rate on the material used as a patterned mask) is also very high for quite soft materials. It is then possible to use many different materials as a masking pattern to protect the region not to be etched—such as the simple resist used in lithography, thus making the process very fast and easy—or other more complex materials with other potential functional properties. Many authors have referred to the possibility of realizing tens of micrometer high SHS in silicon [61,62,63,64,65,66,67,68]. Microfabrication techniques development allowed the realization of SHS structures directly on thin substrates (few tens of micrometers), through which it is then possible to realize holes between the pillars. To obtain this, from a fabrication point of view, it is usually easier to reverse the process with respect to how we presented it here, i.e., to realize first the holes through the substrate material and only later the pillars. In this case, a superposed combination of successive steps of optical lithography defines the desired patterns into selected masking materials or resists on the thin substrate, usually supported on a rigid thicker support wafer, and finally, the DRIE engraves both structures—holes and then pillars—in the device after selective removal of the specific holes mask between the two etching steps. Alternative processes have been demonstrated, which realize the two independent lithography and DRIE processes on the two sides of the thin support material [63,69,70].

Reactive ion etching processes can actually allow the realization of SHSs on other materials, as in the work of Accardo et al. [71]. They demonstrated the possibility to realize ordered or disordered SHS by reactive ion etching of poly-methyl-methacrylate (PMMA). They used a hard mask of gold defined by negative optical lithography to pattern a PMMA layer coated on a hard substrate and subsequently etched the PMMA with a plasma of a combination of Ar and O_2_ gases. They found that proper combination of gases and power given to both the coil and the platen is fundamental to achieve the good aspect ratio of the final structure that is necessary to reach the superhydrophobic state since this process is much less selective than silicon DRIE. In silicon DRIE, as mentioned before, the chemical affinity of fluorine radicals generated by ionization of fluorinated gases precursors in the plasma is so high that very deep profiles can be dug in the substrate using relatively soft mask materials as well. On the contrary, oxygen plasma etching on rather strong plastic materials such as PMMA is less selective, leading to a concurrently high consumption rate of the masking material, even when using heavy metals such as gold. The power and the gas composition optimizations are then fundamental to achieve the optimal etching selectivity and to optimize the final aspect ratio of the structure. Moreover, appropriate tuning of the etching parameter can induce a nanotexturing of the surface that further improves superhydrophobicity [72].

### 3.2. Bottom-Up Fabrication Methods and Combined Approaches

**Figure 3:**
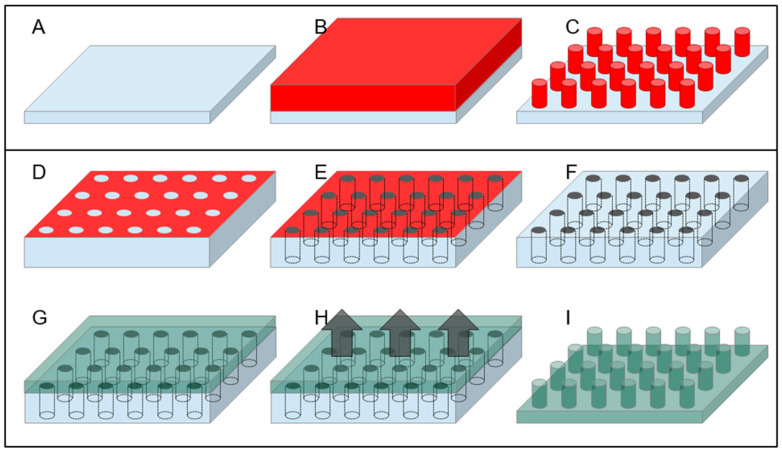
Schematic representation of the most common bottom-up approaches to realize a patterned device with a 3D profile. The simplest way is to use a resist as a patterning material, i.e., to cover a supporting substrate with a resist which, after exposure (by UV light, electrons, or X-ray) and subsequent development, leaves a 3D structure on the substrate (**A**–**C**). The advantage of this technique is the possibility to combine pillars and structures of resist with basically any type of substrate, which can be chosen to be transparent, extremely thin (such as Silicon-Nitride-suspended membranes), have very low background signal for Raman spectroscopy (such as Calcium Fluoride for example), or any other desired property. Panels (**D**–**I**) describe another common and versatile bottom-up approach to microfabrication: using top-down techniques, a mold in a hard material is realized, which has a negative pattern of the structure to be realized (**D**–**F**); on this mold, a liquid precursor of the material to be realized is poured and lately hardened (by photo- or thermocuring typically, panel (**G**)). The hardened material is then delaminated from the substrate (**H**) to lead the final structure, which can then be replicated several times at a very low production cost (**I**).

A rather different approach, as mentioned above, is represented by the use of the so-called bottom-up technique for the fabrication of SHS. The simplest approach is perhaps the use of the lithography resist as a material to realize directly SHSs since many optical and electron beam resist can be spun at variable thickness and, after exposition and subsequent development, can produce a patterned structure with vertical sidewalls, as illustrated in Figure 3A–C. Nevertheless, many resists, as they are polymeric material, can undergo degradation with time and swallowing upon absorption of environmental humidity. Moreover, if the substrate has to be used in temperature-controlled experiments, the situation can be even worse since increasing temperature in patterned resists can lead to their softening with effects that go from the rounding of the features’ sharpest angles down to the complete meltdown of the structures. A rather convenient material to avoid these adverse circumstances is SU8, a negative optical resist—sensitive to electron beam as well—based on epoxy resin, which becomes photocured when exposed to light. The material obtained is rather hard and can be further hardened if thermally cured at temperatures up to 300 °C for a few minutes. The resulting material is particularly resistant to chemical attack and has the further advantage of being transparent to visible light. SU8 then, when deposited and patterned on a transparent substrate, can provide a completely optically transparent superhydrophobic device [73].

Another interesting material for superhydrophobic application is polydimethylsiloxane (PDMS). PDMS is a relatively soft, biocompatible polymer, which has already found a lot of different applications in superhydrophobicity, owing also to the intrinsic hydrophobicity of its surface [74,75]. An important advantage of this material is its versatility, being possible to machine it with top-down and bottom-up approaches, or a combination of the two. From one side, it has already been demonstrated the possibility to machine a bulk surface of PDMS by plasma etching or laser machining to obtain a SHS; from the other side, a great plus of this material is the possibility to be molded at basically any shape in a very easy way [76,77]. Starting from a hard negative mold, which can itself be realized in many ways—such as the use of micromachining or laser ablation on many different hard substrates—it is possible to realize a final shape of PDMS following a well-known and easy-to-replicate streaming of steps. It is enough to pour the liquid PDMS precursor on the hard mold and bake it, typically after degassing the liquid under a low-vacuum pumping to remove gases dissolved in the precursors, to obtain a negative replica of the mold that can be easily peeled off (as shown in Figure 3D–I). Superhydrophobicity of this surface can further be controlled by changing the nanometer-scale topography of the surface with postprocessing, which has been demonstrated in the case of using plasma treatment or laser annealing/etching of the surface. Finally, another important property of PDMS is its intrinsic biocompatibility [78]. Polytetrafluoroethylene (PTFE) is another potentially interesting polymeric material, and SHSs made of PTFE have already been obtained by using chemically induced nanotexturing of the surface [76] or laser ablation techniques [77]; moreover, this material has the peculiar advantage of being already extremely hydrophobic on its own, leading to micro/nanostructured patterns displaying strong superhydrophobicity without the need of any further coating.

## 4. The Hydrophobic Coating of Surfaces

When dealing with fabrication of SHSs for manipulation of biomaterial, and more generally for any kind of application, it is necessary to consider an important aspect: as discussed, superhydrophobicity emerges from the interplay of intrinsic hydrophobicity of the surface and its geometry. In general, as for most of the previous examples, it is convenient to start from a material that is hydrophobic in itself. This is the case for many polymeric materials in general, and specifically, the SU8 mentioned above.

In some cases, the specimen is not hydrophobic enough, or not at all. This is the case of the silicon-based and of all metallized samples, which covers a wide class of applications. In such cases, since contact angle is a surface property rather than a bulk one, and wetting depends on the outermost atomic layers of the substrate material, hydrophobicity can be induced with an appropriate surface coating or a dedicated surface treatment.

Plasma treatment, to consider the latter case, is a well-known mean to modify the wetting properties of a surface in many materials. It can be used as a stand-alone method for obtaining SHSs, or alternatively combined with a preexisting patterned surface. The mechanism underlying this effect actually relies on a chemical transformation of the outermost layer of the material but is frequently combined with a surface modification induced by differential erosion rate to different constituent compounds, which leads to an increased roughness, frequently at the nanometric scale, as indicated at the end of the previous section. The effect on the material of the plasma treatment depends mainly on the chemistry of the interaction of the material’s surface with the gas precursors used in a plasma. Oxygen plasma, for example, is known to increase hydrophilicity on silicon and its compounds and is commonly used to improve water fluidity and decrease the possibility of void formation in microchannels for many in situ experiments. On the other side, its effect on PMMA seems to go in the opposite direction, and it has been used to increase hydrophobicity in combination with the increase in surface roughness as well [79,80].

**Figure 4:**
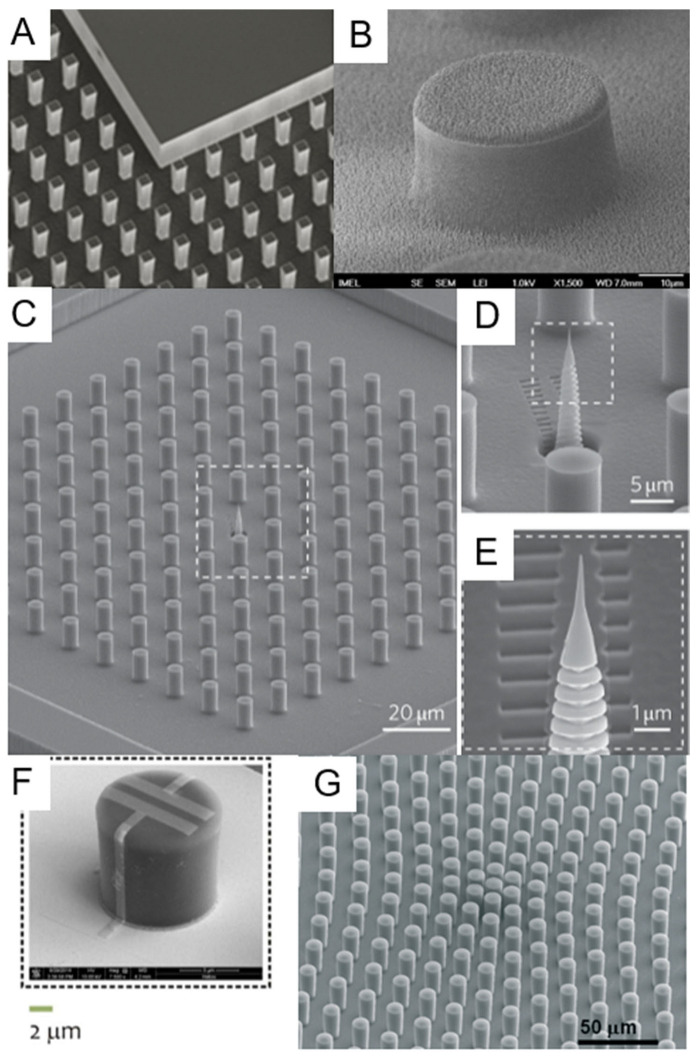
Panel (**A**) shows a silicon-based superhydrophobic concentrator having at its center a hydrophilic area to collect the concentrated material. Panel (**B**) reports a scanning electron microscopy (SEM) image of a SU8 pillar of a SHS. Plasma etching was used to create a “dual scale topography” to enhance the superhydrophobic behavior of the surface. Panel (**C**) (with higher magnification details in (**D**) and (**E**)) reports a SEM image of a silicon-made superhydrophobic concentration device having at its center a plasmonic focusing tip to enhance the local electric field for few-molecule Raman signal excitation. A SEM picture of a pillar realized on silicon is shown in (**F**), which has a dual-electrode sensor fabricated on its top and contacted for electrical measurements. Panel (**G**) shows a SEM image of an arrangement of SU8-grown pillars, in this case, realized on a silicon nitride membrane for sample optical and X-ray transparency, having a positive pillar density gradient toward the center to force the suspended drop in this position while maintaining the superhydrophobic state. (**A**) Reproduced with permission from ref. [116]. (**B**) Reprinted with permission from ref. [87]. (**C**–**E**) Reprinted with permission from ref. [66]. (**F**) Reprinted with permission from ref. [67]. (**G**) Reprinted with permission from ref. [73].

In some cases, it can be beneficial to the achievement of an improved superhydrophobicity to further increase material roughness at a smaller scale, even in a disordered way, to obtain a final surface with a sort of hierarchical structure displaying a nanometer-size disordered roughness superposed to a micrometer-scale ordered array. Plasma treatment can be used as well in this direction, providing a nice example of integration of the two approaches, the bottom-up and the top-down ones. The local nucleation of harder points during the plasma attack of a surface can be used to induce a locally controlled roughness [81]. This has been achieved on many different surfaces, starting from silicon [82,83], where it can be seen as a modification of the process used to produce the so-called “black silicon” [84,85,86]. Marquez-Velasco et al., have realized an SU8 microscale pattern and, by adding a subsequent plasma etching step, patterned the structure with what they call a “dual scale topography” (Figure 4B) [87]. They realized a nanotexture of the surface of the micropattern, thus obtaining a hierarchical structure improving the hydrophobic properties of the surface. In an analogous way, a similar result has been obtained on PMMA [88]. Salapare et al., demonstrated the possibility to induce stable and durable superhydrophobicity on polytetrafluorethylene (PTFE) by oxygen plasma treatment [89], while Correira et al., demonstrated analogous possibility on poly(l-lactic acid) electrospun membranes [90]. Cortese et al., demonstrated an analogous dual roughness structure in PDMS by combining the standard bottom-up approach to produce a micropatterned surface from a silicon hard mold by thermal-induced curing of PDMS with a post-lithography plasma roughening at nanometric scale of the surface [91]. Another interesting approach was used by Chen et al. [92]. In this paper, the authors combined the patterning of a surface with a resist and used conformal coating by parylene deposition. The uppermost parylene layer was then nanotextured with plasma etching using a nanotextured self-assembled mask [93]. Actually, protocols using plasma-assisted nanotexturing have been demonstrated that could, in principle, be transferred to many different types of substrate materials [93,94].

Another way to obtain a surface able to suspend the drop in the Cassie–Baxter state from a patterned hydrophilic material is to put a hydrophobic surface finishing to make the structure superhydrophobic as a whole. Hydrophobicity of any material in this case can be induced with an appropriate surface coating whose thickness can be reduced down to the single molecular monolayer. A common solution that has been employed frequently to increase hydrophobicity of a surface is coating with fluorocarbon compounds. There are different techniques to apply such a coating, including the deposition of perfluorinated polyethylene by plasma-induced deposition from a gas precursor and the creation of monolayers of perfluorinated hydrocarbon molecules on the surface by self-assembly [95,96,97].

In the first case, an example is represented by the plasma-assisted deposition of PTFE (poly-tetrafluoroethylene) material by using a fluoroalkane precursor in the gas phase, usually perfluorocyclobutane (chemical formula C_4_F_8_). This is basically the “deposition” step of a DRIE process, but used in a stand-alone way, without any interleaved etching step. This method allows the deposition of a perfluorinated polymer layer whose thickness can be controlled down to the 1–2 nanometer range. Examples of the second way are the creation of a self-assembled monolayer of perfluorinated molecules on silicon and silica-based materials by chemical interaction in a vapor phase deposition process using perfluorodecyltrichlorosilane (FDTS) [98] or by self-assembly of a monolayer of tetraethoxysilane (TEOS) from a liquid solution [99]. This molecule has a reactive termination that, upon diffusion in vapor in a vacuum chamber with a coreactant (water in this case), binds to the silica surface. The other side of the molecule is a perfluorinated decyl termination that creates a monolayer of hydrophobic terminations. Both methods are promising with specific differences. The plasma-assisted deposition is prone to inhomogeneities on the surface, and hence, requires a thicker layer of material to be deposited to be sure of the result; it is also quite general, i.e., it can be applied to any type of surface and material. On the contrary, the vapor phase deposition method to create self-assembled monolayer has a much better precise thickness control and uniformity of result, but it requires a specific termination optimized for adhesion to a particular material or a family of similar materials. Switching to different substrates requires either the development of a new molecule with specific binding termination, or the deposition of an intermediate adhesion layer.

## 5. SHS for Manipulation of DNA and Proteins

SHSs have been largely used to manipulate and control the motion and the deposition of water drops [100]. This led to the study of another important dynamics of the droplets: their evaporation [101,102,103]. It has been demonstrated that the presence of an underlying SHS alters the evaporation dynamics of a water drop [104].

In case the drop is not simply made by pure water, but rather, by an aqueous solution containing some dissolved solute, the evaporation process leads to interesting developments. Shrinking of the solution drop due to volume loss creates a stress on the drop structure, due to the fact that keeping the same contact line for a drop of reduced volume implies the maintenance of an always-growing angle of contact on the substrate. The only way the drop has to compensate for this excessive contact angle is to regress the contact line, a process that can only proceed jumping from one pillar of the SHS to the neighboring one (see Figure 1C for an illustrative picture). De Angelis et al., for example demonstrated that this leads to a concentration of the solute inside the increasingly small drop (Figure 4C–E). In this way, it is possible to concentrate a very small amount of dispersed chemicals in water into a very small spot, leading to the possibility to detect dispersed substances at a very low concentration [66]. In this paper, the authors realized a silicon-based, DRIE-milled SHS decorated on the top of the pillars with a silver film roughened at the nanometric scale. By doing this, they combined two different purposes of the coating film: apart from serving as a selective mask for silicon substrate etching in DRIE, it also works as a sensing substrate. They used a positive resist lithography to define a regular pattern of circles on the surface of a silicon wafer. Then, the silver film was realized using a self-assembly, bottom-up, electroless deposition chemical approach based on the decomposition of a liquid precursor (silver nitrate in water) supported by a reducing agent (HF). This reaction is catalyzed by the underlying silicon substrate; hence, it takes place only in the holes left open in the resist by lithography. The pattern was then etched down to several micrometers to realize the morphological structure of the SHS [105]. The sensing mechanism implemented on the device by the silver film is based on the phenomenon of Surface-Enhanced Raman Scattering (SERS) [106,107,108]. In SERS, the normally quite weak Raman signal from a molecule is amplified in correspondence of structures or defects of a conductive substrate material due to the local concentration of the electromagnetic field at optical frequencies in nanometric field “hot spots”, which allow the detection of a very small amount of material, down to the single-molecule level. In this respect, electroless-deposited noble metals have demonstrated to be efficient and effective SERS active substrate [109,110]. In the work of De Angelis et al., the authors demonstrated the capability to overcome the diffusion limit in the detection of highly diluted solutions of different molecules by using the concentration capabilities of an SHS device [111]. The solvent evaporation of a drop of diluted material placed on the SHS leads to a strong concentration of the originally diluted molecule to detect in an area at the center of the SHS. In this case, since the final intention is to analyze the molecules once concentrated, the authors integrated into the superhydrophobic device the capability to enhance Raman scattering by using the mechanism of SERS. Ebrahimi et al., demonstrated an analogous application towards overcoming of the diffusion limit in analyzing liquid solution of diluted DNA [112]. In this case, the SHS was realized by nanostructuring the top of an array of Nickel electrodes used for the detection of DNA. Upon evaporation of water from the deposited drop, attomolar detection of DNA filaments in solution was demonstrated. Other implementation of superhydrophobic concentrators have been proposed in combination with SERS detection of dissolved molecules, but they were based on disordered self-assembled structures without an ordered pattern [113,114]. On the other side, De Ninno et al., proposed an analogous solute concentrator for the detection of proteins. In this case, they used a silicon-based RIE-etched structure, made superhydrophobic via silanization with trimethylchlorosilane to concentrate the evaporation residuals in a central area of the device, where an array of gold nanoantennas was fabricated to enhance the IR response from the sample [115]. A similar device was also used as concentrator of biomaterial for X-ray diffraction analysis and is shown in Figure 4A [116]. Ren et al., used a PDMS-made SHS treated by CF_4_/O_2_ plasma to increase superhydrophobicity as a concentrator of DNA for high-dilution detection [117]. Other authors have obtained an analogous result on DNA or other molecules using a disordered SHS [118,119].

These papers on DNA concentration led to further development in the manipulation of biological materials while observing the deposition of a physiological solution containing biological materials of interest—might it be biomolecules, or cells, or part of cells—on an SHS of this type in the Cassie–Baxter state. Upon evaporation of the drop, recession of the contact line of the drop on the surface causes the deposition of part of the dissolved biomaterial in fashions that are of interest for many applications: it is possible to obtain regular organization of DNA bundles and filaments, stretched cells membranes, and protein superstructures.

For example, DNA stretching of small bundles of double-helix DNA filaments has been demonstrated. This technique relies on the shear-force-induced stretching of the filaments across the interpillars gap during the evaporation of a drop of physiological solution containing a dispersion of DNA filaments. It is during the jumps that the contact line of the drop on the SHS performs from one pillar to the next during shrinking that DNA bundles and filaments that are attached to the top of the pillars get stretched across the interpillars gap by the shear force induced by the liquid movement (Figure 1C) [69].

The optimization of this process requires integrating surface functionalities into an optimized fabrication process in order to obtain pillars having a gold layer on top. This is because of the increased adhesion of DNA filaments to the top of the pillar due to electrostatic interaction since the final purpose of the device is to improve the production of self-induced stretched DNA filaments and bundles across the interpillar gaps during the recession process of the water contact line. To this purpose, a combined deposition of multilayered metals in high vacuum after a positive optical lithography creates a layer of surface gold in correspondence with the top of the final pillars, which is protected by an upmost layer of Chromium. Gold is a rather soft material and gets etched by DRIE of silicon in a relatively fast way. On the contrary, Cr is much more resistant: few tens of nm of Cr can protect a substrate area from etching when drilling silicon up to few hundreds of micrometers in depth. Nevertheless, since the two metals can be deposited in sequence in the same process, it is easy to combine the final desired device property—pillars whose top is Au-covered—with the necessity to mask pillars from etching with a hard Cr mask [120].

**Figure 5:**
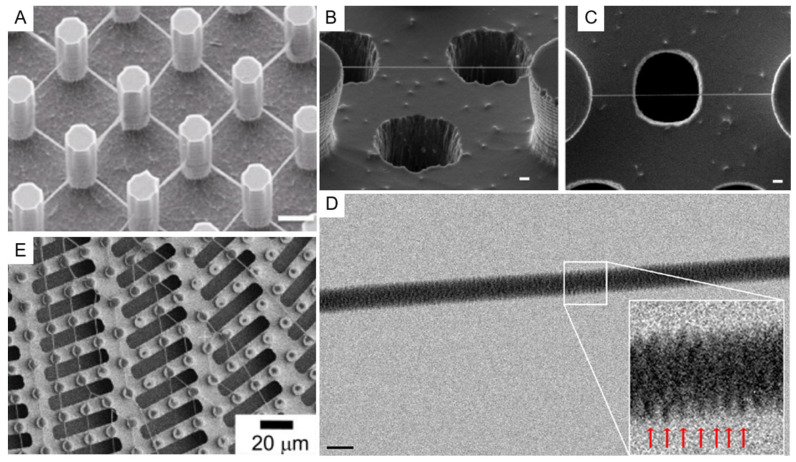
Silicon-based SHSs for material stretching across pillars. Panel (**A**) shows a SEM picture of a deposition of Deoxyribonucleic acid (DNA) bundles stretched across a silicon-made SHS. Authors demonstrated the possibility to have control on the orientation and vertical position of the created bundles. Panels B,C: side (**B**) and top (**C**) view of a silicon-based SHS with DNA bundles stretched across the interpillar gaps. It is then possible to obtain High-Resolution Transmission Electron Microscopy (HRTEM) images of the bundles that show a periodic structure of the filament, which is coherent with known values of DNA helical structure (shown in (**D**), with magnified view in the inset). Panel (**E**) displays a SEM view of a microfabricated silicon SHS, perforated to allow TEM analysis of the deposited material. The picture shows the formation of fibrils resulting from the evaporation in the superhydrophobic state of a physiological solution containing Tau441 proteins stretching across the pillars. Elongated holes between the pillars assure complete transparency to the electron beam even upon sample tilting. Scale bar in panel (**A**) = 5 μm; in (**B**) and (**C**) = 1 μm; and in (**D**) = 20 nm. (**A**) Adapted from ref. [123]. (**B**–**D**) Adapted from ref. [69]. (**E**) Adapted from ref. [154].

A further development of this type of approach was reported by Ciasca and colleagues [121]. In these papers, the authors demonstrated the capability to control direction and position of the stretched filaments by properly designing the shape of the pillars used for the SHS. By using an asymmetric shape for the pillars, it was possible to induce the jumps from one pillar to the other at specific sites, obtaining a quite-regular array of DNA stretched bundles oriented along specific directions [122]. A further refinement of these techniques was obtained by the same group with control of the level of hydrophobicity of the surfaces. They demonstrated a kind of intermediate state between the Cassie–Baxter and the Wenzel ones, in which there is a partial impalement of the drop on the pillars, but globally retaining a layer of air between the drop and the bottom of the structure. They proved to be able to control the vertical positioning of the web of DNA bundles stretching across the pillars, thus adding a further degree of control on the process, as demonstrated in Figure 5A [123].

Isolated and well-organized deposition of self-assembled free-standing DNA filaments and bundles have proven to be a valuable tool for many different applications. Apart from representing a support layer over which other molecules can be deposited, it has been demonstrated the possibility to perform Raman experiments that are capable of obtaining the vibrational signature spectrum from DNA with a strong suppression of any interfering signal coming from the support substrate, due to its relative distance from the suspended material. Scanning Electron Microscopy (SEM) has been extensively used to characterize these DNA structures to obtain information on their size and shape, and on the morphology of any material deposited on them [124,125].

Another interesting application concerns the study of the mechanical properties of these DNA bundles. There is a growing interest among the biomedical community on the mechanical properties of DNA filaments, as there is evidence pointing at a correlation between this property of DNA and the eventual alteration of its structure associated with heavy metal environmental pollution and other conditions leading to DNA alteration, which are of interest in the study of a number of diseases such as some types of cancer. Self-organized bundles of DNA suspended on SHSs have been characterized by Laser Doppler Vibrometry. Basically, the DNA filaments are treated as bioinspired analogues of nanomechanical oscillators. Standard MEMS-type fabricated nanoresonators have been recently developed for applications as ultra-precise vibrometers and ultra-high-precision nanobalances capable of measuring a weight down to the single-molecule level. Due to the intrinsic higher softness of the biological material, DNA nanovibrators cannot achieve these performances; nevertheless, researches have demonstrated that it is possible to deduce information on the DNA stiffness from the measurement of the vibrational spectrum of such DNA bundles and to correlate such information with the presence of intercalant dopant altering the DNA structure [126]. A similar but different approach is the one used by Borin et al. [127]. In this work, there is no stretching of DNA filaments across the gaps between pillars in a silicon patterned SHS, but rather, they produce a self-assembled monolayer of DNA molecules on top of each pillar. The entity of such layer is then detected by vibrometry in vacuum as well. The difference in this case is that the silicon pillar itself is the resonator, and the change of its intrinsic vibration frequency in vacuum gives the signature of DNA deposition [128,129].

The interest in this type of SHSs then shifted onto proteins, protein assemblies, and superstructures. The opportunities offered by the evaporating drop on an SHS are different. First of all, it is possible to study the interaction of proteins with DNA. Analogously to what is done on simple DNA or DNA doped with intercalants, DNA–protein complexes can also be stretched across the gaps of the SHSs, leading to the possibility to collect structural information on the overall assembly created by the interaction of the two components. An example has been demonstrated for the case of rad51, a DNA-repairing agent active in many biological systems, which has been observed creating ordered complexes on DNA-stretched filaments [120]. Subsequently, it has also been examined the possibility to obtain self-assembled structures from proteins only, without the supporting function of DNA filaments.

Gao et al., managed to obtain crystallization of lysozyme using a proteinaceous superhydrophobic material [130]. In this case, a self-aggregate protein complex, properly functionalized, is used as superhydrophobic substrate, lacking an ordered patterning [131]. On such substrate, the authors demonstrated the ability to manipulate the low-concentrated protein solution to induce their crystallization for structural studies. Shiu and Chen instead proposed using a irregularly patterned nanostructured surface that has the possibility to switch its wetting state from very high to very low wettability as a method to selectively spot proteins on the surface. The surface could be obtained by producing a PTFE-like material deposited by a liquid spin-coating precursor on an indium-tin-oxide-coated flat substrate and subsequently randomly nanostructuring it by means of an oxygen plasma treatment [132].

Many proteins tend themselves to naturally form aggregates, which are of great interest in many fields of application. Spider silk, for example, is the result of the aggregation of proteins naturally produced by spiders. Due to the very high mechanical strength, it has raised a lot of interest and attracted a great deal of research for decades, which was more recently fostered by the interest for the application of biomaterial for surgery and scaffolding due to its biocompatibility. Nevertheless, the mechanisms underlying the constituent proteins’ self-aggregation to form the silk has never been fully reproduced in a reliable and biocompatible way [133,134,135]. Recently, the use of superhydrophobicity has led to an important step forward in the control of artificial silk production. Gustafsson et al., reported the possibility to obtain self-aggregation of filaments starting from a solution of silk precursor proteins by deposition from a drop suspended on an SHS [136]; the SHS production was also based on a DRIE process on silicon to define a regular pattern of pillars, followed by a surface treatment by plasma-induced deposition of PTFE, analogously to the DNA examples mentioned above. The substantial difference in this case was due to the combination of a controlled movement of the drop on the substrate with the drop evaporation. This has led to the production of well-organized filaments of spider silk proteins stretched between the different pillars. Similar results have been obtained on polymeric structures, but with a lesser degree of control due to the use of a disordered structure [137].

It is also the possible to obtain stretching of protein fibrils preaggregated in the suspended solution. It has been clarified in recent years that many important degenerative diseases such as Alzheimer’s are associated to the formation in the patients’ body of protein fibrils due to anomalous aggregation of proteins normally playing an important role in brain physiology. The study of the structure and properties of these fibrils has received a great deal of attention in order to understand the biomolecular mechanism associated to their self-aggregation, by means of many different techniques spanning from Raman to Transmission Electron Microscopy (TEM), Atomic Force Microscopy, X-ray diffraction, and nuclear magnetic resonance [138,139,140,141,142,143,144,145,146,147,148,149]. This has triggered the interest in the possibility to have suspension of self-assembled fibril filaments across gaps in SHSs, as performed by Moretti et al. [150]. In their paper, the authors proved that it is possible to suspend stretched filaments of amyloid fibrils obtained as aggregation of lysozyme proteins on silicon-based, DRIE-etched superhydrophobic devices. Moreover, they proved that it is possible to obtain a very clean and clear Raman signal to analyze the structural properties of these fibrils without having to deal with any relatively strong perturbing signal coming from an underlying substrate. A similar type of aggregation was observed on other proteins as well [151,152,153].

The application of these devices to the study of self-assembly of protein has even reached the stage that it is possible to induce filament aggregation of proteins originally nonaggregated and dissolved in the solution of the suspended drop. Zhang et al., proved that the liquid Marangoni convection inside a drop suspended on the SHS under a temperature gradient can induce the fibrils aggregation. These protein superstructures can aggregate out of a dilute solution in this drop-size-suspended microreactor and create a stretched and deposited structure on the device after complete solution evaporation. These structures have provided interesting insights into the aggregation process of the proteins once studied by Raman and X-ray spectroscopy [154].

Another important biological structure that has been studied with the help of these devices is the exosome. Exosomes are nanometric-size micellar agglomerates having a lipidic bilayer outer membrane. They are attracting a growing interest in applied biology and biomedical studies due to their important role in cellular intercommunication, with very important implications in cancer development [155]. For this reason, an increasing number of studies is devoted to understand their structure and properties [156,157,158].

With these kinds of devices, virus nanofilaments self-aggregation were observed as well as localized X-ray diffraction information, and Raman spectral analysis from this self-assembled nanostructures was obtained [159,160].

A crucial aspect of these SHSs, which has been pursued for many different applications in biomaterials characterization, is the low perturbance effect given by the substrate material for the different types of characterization employed. We already mentioned among the fabrication methods several ones leading to the production of optically transparent SHSs. As a further example, the possibility to grow SHS made by an amorphous material such as SU8 on amorphous silicon nitride membranes only few tens of nanometers thick provided samples that are not only optically transparent, but whose transparency extends to the domain of X-rays. Such surfaces have demonstrated their usefulness in the study of exosomes and amyloid fibrils after self-assembled deposition upon solution precipitation of these materials by means of synchrotron X-Ray characterizations [72,161,162,163]. Since X-ray scattering from deposited samples is analyzed, it is important for the substrate to provide weak or no contribution to the scattered signal. This is the motivation for the use of low-Z amorphous materials such as polymers, as done by those authors in their studies. Another important example is represented by the use of silicon-based microfabricated devices combined with the possibility to obtain perforated samples in correspondence of the suspended material. Figure 2D–I describes a schematic approach on how this can be implemented, while Figure 5B–E show some example of application of these structures for characterization of DNA and proteins self-aggregates. This leads to the possibility to perform TEM characterization of the suspended filaments of biological material without the perturbing effect given by the randomly distributed signal generated by an underlying substrate. Consequently, High-Resolution TEM (HRTEM) images of bundles of DNA have been obtained showing their structure and the periodicity of the chains [69,70,120]. Optimization of the protocols has led to the possibility to image DNA down to the limit of the single filament [63] and this approach allowed researchers to deduce important structural information on DNA filament after interaction with proteins and other intercalants [120].

## 6. SHS for Manipulation of Cells and Cellular Derived Structures

Another type of biomaterial for which the peculiar properties of SHSs have been exploited is represented by the cells and cellular structures, and also for cell growth and culturing. From one side, superhydrophobicity can be exploited as for DNA and similar materials to suspend part of, or entire, cells with the aim of obtaining suspensions of these materials across the structure pillars.

**Figure 6:**
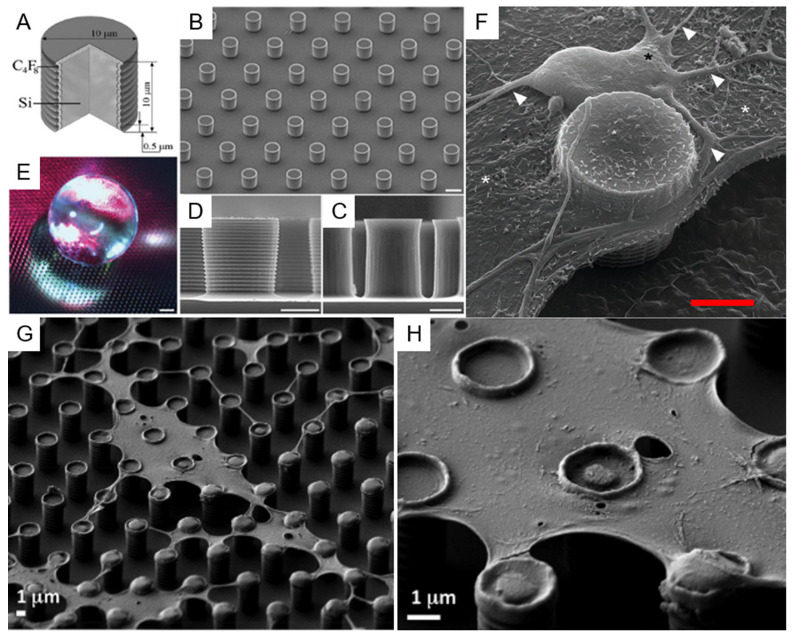
SHSs for cells and cellular membranes deposition. (**A**) Structure of a DRIE-made silicon pillar and (**B**) experimental realization with a regular arrangement. (**C**) and (**D**) show the lateral profiles of two different implementations: in (**C**), the smooth side surface of the pillar is obtained by using a single etching step; in (**D**), standard DRIE process with alternation of deposition and etching phases results in the typical saw-teeth profile, an effect usually termed scalloping. This latter lateral profile has been proved to be important to promote adhesion of cells and is preferred for this application. (**E**) Image of a water drop suspended on an SHS. (**F**) SEM image that shows the presence of a large neuron (black *) with its multiple neuritic processes (arrows) spreading on top of a flat glial cell monolayer (white *), suspended on a silicon-made SHS identical to that shown in (**B**). (**G**,**H**) SEM tilted views of a silicon-made SHS over which cellular membranes have been suspended and stretched. Scale bars: 10 μm in (**B**), 5 μm in (**C**,**D**,**F**). (**A**–**E**) Reprinted with permission from ref. [64]. (**F**) Adapted from ref. [64]. (**G**,**H**) Reprinted with permission from ref. [120].

As an example, such an approach was successfully used for the suspension and stratification of cell membranes patches. In this case, the authors introduced a modification of the superhydrophobic pattern and used a different surface coverage with respect to the one used for the studies on DNA and proteins. Silicon structures without metallic coating have proved to perform best, leading to the possibility to stretch on the SHS entire portions of cells membranes, with a mechanism very similar to the one involving DNA, i.e., as a result of the shear forces created by the liquid solution regressing from one pillar to the next upon water evaporation and drop shrinking, as shown in Figure 6G–H. The main point here is that, due to the different geometry of the structures to be deposited with respect to filaments of biomaterial, since these membrane portions are more planar and more similar to a two-dimensional (2D) structure than a 1D, the best performances are found in a regular pillar pattern made of a homogeneous square array, rather than in an arrangement of circular concentric lines. More in detail, in the first case, the fact that the structures to be deposited do not have an axial symmetry but are rather planar implies that a uniform geometrical arrangement better stretches cells and membranes across the gaps in the structures. On the contrary, referring to the DNA filaments, they tend to stretch more efficiently across gaps between pillars oriented in a radial way with respect to the axis of symmetry of the drop, thanks to the stretching mechanism connected to the recession of the water contact line on the substrate.

Other important structural point about SHS is that, in this application, a smaller structure spatial periodicity is required as well to be able to stretch efficiently the membranes. This occurs because of the relatively smaller lateral size of the obtained membrane’s portions with respect to the length of the filaments. Here is a demonstration, as mentioned in previous paragraphs, of the versatility of the design and implementation offered by MEMS-type microfabrication techniques for the manipulation of biomaterials. Their design can be easily modified, and it is possible to produce many different designs and shapes of the three-dimensional (3D) structure, combined as well with many different surface coatings, which can be adapted to a large variety of biostructures to be manipulated, suspended, and analyzed.

Further, in this case, it was possible to combine this approach with the realization of the SHS on few tens of micrometers thick perforated substrates. These devices have allowed the possibility to obtain in situ HRTEM images of portions of plasma cells’ membranes, leading to the possibility to obtain high-resolution images of transmembrane proteins [120].

An interesting evolution of this approach is represented by the work of Malara et al. [164], in which the authors realized a SHS device by creating SU8 micropillars on a silicon substrate. To control the position of the drop once deposited, they deformed an originally periodic pattern of pillars to obtain an increasing pillar density while proceeding towards the center of the device, thus introducing a positive gradient of the surface coverage *Φ* while proceeding toward the center of the device (Figure 4G) [165]. The authors realized special, two-electrodes sensing devices on the top surface of specific pillars. These sensors were made in a planar configuration of two gold electrodes facing each other, covered by a thin layer of a conductive polymer—poly(3,4-ethylenedioxythiophene) polystyrene sulfonate (PEDOT:PSS), as shown in Figure 4F [166]. Once a drop of a solution prepared from blood clinical samples was placed on the substrate, these devices, by studying electrical conductivity in the sensors, demonstrated successful cancer prediction and tumor risk assessment capability [67,68]. Such results find similar applications in devices for tumoral cell detection and isolation via patterned micro/nanostructures that instead do not exploit the superhydrophobicity [167]. There has also been a great interest toward the study of the effective growth and proliferation of living cells on such substrates, not just the manipulation of part of cells or of their derivates as in the above examples.

One of the explored directions is represented by the use of patterned structures devoted to understanding their effect on cell growth. Neuronal growth and proliferation have been demonstrated to be affected by the direction of grooves placed on the substrate [168,169], while more elevated silicon-based structures have proven the possibility to produce 3D layering of the cultured cells [170,171]. Despite the undoubted interest raised by these experiments, they do not make use of superhydrophobicity or of any control on the surface wetting.

On the other side, other groups have worked to understand the effect of wetting on cell culturing and proliferation. Studies on the viability for cell growth of SHSs have been realized in different materials, but generally these studies refer to self-assembled disordered patterns [172,173,174,175,176,177,178,179,180].

Oliveira et al., have proposed the use of a patterned superhydrophobic device based on porous material as an easy and high-throughput system for assessing the materials best conditions as substrate for tissue engineering and scaffolding applications [181]. The substrate was prepared via a controlled modification of polystyrene surfaces through a combination of exposition to UV light and to Ozone, according to a protocol previously developed for microfluidic applications [182]. The protocol was enough versatile to allow to switch the surface properties from completely superhydrophobic to superhydrophilic by just changing the process parameters. Combination of such approaches with surface patterning and controlled deposition of cells can also lead to a precise and controlled deposition of single cells or cells agglomerate [183]. The same group has proposed the use of SHSs as a platform to screen the biological performance of independent combinations of biomaterials, cells, and culture media [184,185], and as a mechanism for the formation of cell spheroids for their high-throughput parallel screening [186,187]. Extreme wettability microfabricated arrays were designed to assist and optimize cell adhesion and 3D cell environment establishment for tissue engineering and drug screening applications [188].

Another strong field of interest in this direction is driven by the need for substrates to manipulate cell adhesion in sensing. Ueda and coworkers, for example, realized a substrate that is selectively superhydrophobic to isolate patterns of cells deposited in order to reduce interference and mix-up in high-density cell microarrays [189]. Lima et al., extending an approach based on immobilization of cells in hydrogels for target cell delivery [190,191,192], have used SHSs to prepare mesenchymal stem cells isolated from Wistar rats bone marrow in separated noninterfering alginate beads fabricated using an approach involving the jellification of liquid precursor droplets onto SHSs [193].

Another example of how this approach can be modulated to different structures having different applications is provided by the examples concerning the growth of cells. It has been demonstrated that it is possible to grow and culture cells on superhydrophobic structures. Electrostatic interaction has proved to be important in this application as well. In this case, the Si-etched structure is realized without any specific localized coating, such as the one on top of the pillars of the previous examples, but a global Au coating is placed on the entire sample at the end of the etching process. This particular arrangement has two advantages for this type of applications: from one side, it provides a global coating to the substrate with a material that is more inert and then more biocompatible; secondarily, this gives the possibility to promote a physiological cellular adhesion on all the surfaces of the SHS. It has been observed by electron microscopy that cells can adhere to the nanostructured side walls of the micropillars if kept suspended in superhydrophobicity, also in the Wenzel state, for a defined time necessary to make contact with the surface just immediately after being seeded [64]. Figure 6A–F shows illustrate this application. Table 1 reports a resume of the most important applications relevant to the field described here.

## 7. Outlook and Perspectives

Controlled deposition, orientation, and manipulation of nucleic acids, proteins, small peptides, and cells exploiting the peculiar properties of SHS has demonstrated so far to be a valuable tool for the characterization of structural, biochemical, and physiopathological properties of biomaterials.

From the fabrication point of view, many possibilities are still open in many directions. Silicon-based DRIE-etched samples remain an important standard for preparation of patterned structures, but combination of this technique with a larger platform of surface-finishing methods, such as for example self-assembly of nanoparticles at the surface [7,8] or light-driven selective adhesion of chemicals on the structures [187], would certainly enlarge the spectrum of possible applications. Particles having specific properties, such as SERS active plasmonic field enhancers or organic or inorganic fluorescent nanoparticles could attribute new properties to SHSs that could find new applications, particularly in sensing and in the characterization of biomaterials.

In these silicon-made devices then, due to the high reproducibility of the structures and reliability of the available materials and fabrication techniques, there are large margins of improvement for the few applications made so far in vibrometry [126,127,129]. Micro/nanoresonators have been largely studied so far and weight detection on such resonators have been demonstrated down to the single-molecule level. The combination of this principle with the integration of resonators into SHSs is just at its early stage, but the synergy between the high reliability of MEMS-type devices production and the possibility to locally functionalize them to selectively bind or attract specific biomolecules opens up a large spectrum of interesting applications in biosensing and biodetection in lab-on-chip devices. We are only at the very first stage of this kind of research but a bright future for this kind of detector can already be foreseen for the coming years.

Concerning other materials, still a lot of possibilities are open for exploration. So far, light and electron transparent substrates have been produced combining lithography and new materials. Many others are being explored and, in this case, the combination with tailored functionalization will be of great importance for future applications. Polymeric self-curing materials are particularly promising for their capability to produce low-cost patterned SHSs of different shapes starting from a hard mold. The doping of these polymers with other molecules or particles to impart them specific properties such as electrical conductivity or specific binding properties has just started with few examples [167], but much more is expected to come in the future, as they could also benefit from targeted functionalization. Apart from this, other types of interesting fabrication approaches have been poorly explored in this field. For examples, few researchers have made use of electroplating to prepare SHSs [112], but this is a very versatile, cheap, and easy technique that allows producing different patterns and shapes on basically any substrate, which will turn out to be conductive and could be very easily implemented in electrical circuits for detection, bypassing more complex and expensive fabrication approaches [67].

Surface hydrophobic coating is another very important aspect, as mentioned in Section 4. Plasma-induced surface roughness is certainly a great tool in this respect, as well as vapor-phase deposition or plasma-assisted deposition of hydrophobic layers, but more refined combinations of plasma nanotexturing of deposited coating or self-assembled monolayers of more complex material, such as nanoparticles or functionalized molecules, can be explored [8], with the possibility to obtain surfaces combining hydrophobicity with multiple specific properties aimed at applications on sensing or biocompatibility.

The combination of these preparation methods with new emerging characterization techniques will certainly be extremely beneficial to the field of structural characterization of biomaterial. X-ray, for example, apart from the great potential offered by synchrotron radiation studies [161], can now profit from the development of new advanced tools for the characterization of materials [194]. Raman is another tool of great interest, which has already provided a lot of information from these materials. It can be foreseen that the combination of more developed techniques such as polarized Raman will provide even more information once combined with the oriented biological structures such as DNA and protein fibrils obtained with the self-assembly capability provided by SHSs [195,196]. TEM is another technology that has evolved enormously in the last 10 to 20 years, particularly with the introduction of new aberration-corrected systems that are able to compensate for the intrinsic spherical aberration of magnetic lenses [197,198,199,200,201]. This has led to the possibility to align TEM to high resolutions even at very low accelerating voltages, making it possible to obtain atomic resolution images even on biological materials that, because of the high content of low-Z materials, are easily damaged by electron beams at higher tensions [198,201,202]. The possibility to prepare suspension of biological materials on SHS will then provide other interesting samples for the ever-growing capabilities of the new microscopes. All these techniques will certainly profit from the capability, still to be investigated in depth, of material discrimination provided by SHSs. Superhydrophobicity has shown the possibility to tune the adhesion of different biomolecular components of a mixture making them separately distinguishable [203]. It can be speculated that this sieving effect could be extremely promising for the manipulation of biological materials for in vitro, in vivo, and clinical applications.

Finally, an interesting future development can come from dynamic deposition of the biological material of these samples. While the deposition of suspended biological material from an evaporating drop is limited to the intrinsic properties of the liquid itself, and hence, its retraction speed is solely controlled by the physical properties of the liquid, a better control on the deposition could be obtained by rather using a moving drop. Due to the poor adhesion of a water-based biological solution on a superhydrophobic surface, a small lateral force applied to the drop can push it across the surface at any desired, controlled speed. Such a dynamical exploitation of the drop on a SHS has been rarely explored so far, but it could be an interesting future development to increase the degree of control on the deposited material and on the final arrangement of the biological structures obtained [204,205].

## Figures and Tables

**Table 1:** Resume of the application of regularly patterned SHSs for biological material manipulation, fabrication methods, and relative applications.

Application	Deposited Material	Substrate Material	Coating	Notes	Ref.
Superhydrophobic concentrator	DNA, Rhodamine 6G	Silicon, DRIE-machined	PI-PTFE ^1^	Detection by SERS and focusing of plasmons on tips	[66]
	DNA	Electroplated Ni on SiO_2_ insulating layer	Surface chemical roughening of Ni	Detection of material by impedance spectroscopy	[112]
	apoferritin	Silicon, DRIE-machined	Silanization by TMCS ^2^	Detection by IR spectroscopy, enhanced by nanoantennas on sensing area	[115]
	ferritin	Silicon, DRIE-machined	Silanization by TMCS	Detection by X-ray fluorescence and X-ray phase contrast imaging	[116]
Material stretching across gaps	DNA	Silicon, DRIE-machined with holes	PI-PTFE	Observation of DNA periodic structure by HRTEM	[69]
	DNA	Silicon, DRIE-machined with holes, top Au coating	PI-PTFE	First TEM observation of single isolated DNA molecule	[63]
	DNA+rad51, blood cells membranes	Silicon, DRIE-machined with holes, different Au coatings	Vapor phase deposited FDTS ^3^	Characterization by HRTEM	[120]
	DNA	Silicon, DRIE-machined	Silanization by TMCS	Control of orientation and vertical positioning of filaments	[121,122]
	Spider silk proteins	Silicon, DRIE-machined, reentrant profile	PI-PTFE	First reported structuring of recombinant spider silk on SHS	[136]
	lysozyme amyloid fibrils, PHF6 peptide solution, and Tau441 proteins	Silicon, DRIE-machined with holes	Vapor phase deposited FDTS	Self-aggregation of protein fibrils induced by Marangoni convection. Raman, X-ray diffraction, and atomic force microscopy characterization of depositions.	[154]
	Tobacco mosaic virus	Silicon, DRIE-machined, and SU8 grown on Si_3_N_4_ suspended membranes	PI-PTFE on both types	Creation of crystallized aggregates and stretched filaments, analyzed by X-ray diffraction, atomic force Microscopy, and optical and electron microscopy	[159]
Controlled deposition and aggregation	proteins	Polymethyl methacrylate	Plasma-induced surface roughening	Synchrotron X-ray diffraction of protein aggregate	[71]
	Tau proteins	Silicon, DRIE-machined	Vapor phase deposited FDTS	Induction of formation of protein fibrils in the suspended droplet	[150]
	Estrogen receptor proteins	SU8, supported on CaF_2_ or Si_3_N_4_ suspended membranes	SU8 roughening by CF_4_/0_2_ plasma followed by PI-PTFE	Combined Raman and X-ray diffraction analysis of deposited filaments across pillars and evaporation residuals	[153]
Sensing	DNA	Silicon, DRIE-machined, top Au coating	Vapor-phase-deposited FDTS	Detection of stiffness of DNA and presence of intercalants by laserDoppler vibrometry	[126]
	DNA	Silicon, DRIE-machined	PI-PTFE	Controlled realization of DNA monolayers on pillars, detection via vibrometry on single pillars	[127,129]
	Blood clinical samples	Silicon, DRIE-machined	PI-PTFE, followed by a PEDOT ^4^ coating	Incorporate electrodes for conductivity measurements.Application in tumoral risk assessment	[67]
	Circulating tumor cells	PEDOT, after transfer from PDMS mold		Transparent, mass-producible, and conductive substrate for biosensing applications	[167]
Tissue engineering and cell manipulation	Fibroblasts and osteoblasts cells	Polystyrene	UV light and Ozone selective treatments	Flat biocompatible platform for 3D cell scaffolding	[181]
	Human embryonic kidney and cervical carcinoma cells	HEMA-EDMA ^5^ on glass	UV-induced photopatterning	Selective superhydrophobic patterning to avoid cell interference in different cultured spots	[189]
	Neuronal cells	Silicon, DRIE-machined, completely Au coated	PI-PTFE	Growing of neurons stretched across structured substrate	[64]

^1^ PI-PTFE: plasma-induced deposition of polytetrafluorethylene-like layer. ^2^ TMCS: trimethylchlorosilane. ^3^ FDTS: perfluorodecyltrichlorosilane. ^4^ PEDOT: poly(3,4-ethylenedioxythiophene). ^5^ HEMA-EDMA: poly(2-hydroxyethyl methacrylate-co-ethylene dimethacrylate).
